# The Association between Nutritional Alterations and Oral Lesions in a Pediatric Population: An Epidemiological Study

**DOI:** 10.1155/2021/9992451

**Published:** 2021-10-29

**Authors:** Angela Pia Cazzolla, Michele Di Cosola, Andrea Ballini, Luigi Santacroce, Roberto Lovero, Nunzio Francesco Testa, Vitantonio Lacarbonara, Annarosa De Franco, Giuseppe Troiano, Stefania Cantore, Mariasevera Di Comite, Riccardo Nocini, Lorenzo Lo Muzio, Vito Crincoli, Mario Dioguardi

**Affiliations:** ^1^Department of Clinical and Experimental Medicine, Università degli Studi di Foggia, Foggia 71122, Italy; ^2^School of Medicine, University of Bari “Aldo Moro”, Bari 70124, Italy; ^3^Department of Precision Medicine, University of Campania “Luigi Vanvitelli”, Naples 80138, Italy; ^4^Department of Interdisciplinary Medicine, University of Bari “Aldo Moro”, Bari 70124, Italy; ^5^Department of Clinical Disciplines, School of Technical Medical Sciences, University of Elbasan “A. Xhuvani”, Elbasan 3001, Albania; ^6^AOU Policlinico Consorziale di Bari-Ospedale Giovanni XXIII, Clinical Pathology Unit, Policlinico University Hospital of Bari, Bari 70124, Italy; ^7^Department of Basic Medical Sciences, Neurosciences and Sensory Organs, University of Bari “Aldo Moro”, Bari 70124, Italy; ^8^Section of Ear Nose and Throat (ENT), Department of Surgical Sciences, Dentistry, Gynecology and Pediatric, University of Verona, Verona 37126, Italy

## Abstract

The oral conditions of an individual are the result of different factors, including the subject's genotype, oral hygiene habits, the type of diet, and lifestyle, such as smoking. Nutrition in the first years of life can affect dental health for a long time. To prevent mouth diseases, it is also important to eliminate unfavorable eating behaviour and to amplify protective ones. Eating habits, especially in pediatric age, are an easily modifiable and controllable factor, and diet, in addition to influencing the health of the oral cavity, plays a fundamental role in systemic health. Indeed, a sugar-rich diet can lead to conditions, such as diabetes, being overweight, and obesity. The present research was an epidemiological study, with the aim of highlighting some of the associations between nutrition and oral health. In particular, we studied those lesions of hard and soft tissues that are diagnosed most frequently by dentists: caries, enamel hypoplasia, periodontal disease, and aphotoxic lesions and their associations with nutritional deficiencies and excesses including proteins, vitamin A, vitamin D, B vitamins, and iron and calcium minerals. To perform this study, we recruited 70 patients from the pediatric and orthodontic clinics, aged between 3 and 15 years (y), with mean age of 10.4 y.o. The study was conducted by providing a questionnaire to pediatric patients' (supported from their parents or guardians) on individual eating habits, followed by an accurate oral cavity specialistic examination. The nutritional data were processed by using Grana Padano Observatory (OGP) software, freely provided online by the OPG. The statistical tests performed were the chi-square (*χ*^2^) for independence, and Cramér's V test was used to evaluate the associations between eating habits and oral pathologies. The results showed that certain nutritional vitamin deficiencies and nutritional excesses were associated with definite oral pathologies.

## 1. Introduction

The oral conditions of an individual are the result of various factors, such as the subject's genotype, oral hygiene habits (i.e., toothbrushing), type of diet, and any smoking habits [1–3]. Unhealthy eating habits adversely affect both oral and general health. Nutrition in the early years of life can influence long-term dental health [4–7]. Foods that are harmful to general health can also damage teeth and *vice versa*. Research has been focusing on the effect of nutrition on mucous membranes and hard dental tissues, which can be a systemic effect or a local effect, such as the effect of acidic foods and drinks on the teeth, which can be responsible for dental erosion in patients with good oral hygiene [8–11]. Changing one's eating habits and lifestyle can lead to the improvement of oral and systemic conditions [12–15].

To prevent mouth diseases, it is, therefore, advisable to change the modifiable factors, eliminating unfavorable ones and amplifying the protective ones. Eating habits represent an easily modifiable and controllable factor, knowing that diet, in addition to influencing the health of the oral cavity, plays a fundamental role in systemic health, acting also in the autologous self-renewal stem cell niche, related to the oral mucous membrane trophism [16, 17]. It is well known that a diet rich in sugars can lead to conditions, such as diabetes, being overweight, and obesity [18]. The present research was an epidemiological study, with the aim of highlighting some of the associations between nutrition and oral health.

In particular, our attention was directed towards those lesions of hard and soft tissues more frequently diagnosed by a dental practitioner: caries, enamel hypoplasia, periodontal disease, and aphthous lesions. The nutrients we assessed for excesses or deficits in pediatric patients, based on the frequency of intake of certain foods, were proteins, vitamin A, vitamin D, vitamins of group B, and the minerals iron and calcium. Our main questions were as follows: (1) “What eating habits can influence the onset of these statuses?” and (2) “At what point can a correct diet act on the maintenance of a good trophism of the oral mucous membranes and on the formation and safeguarding of dental structures?”

## 2. Materials and Methods

This research was conducted in collaboration with Elbasan University “A. Xhuvani” (School of Technical Medical Sciences), Elbasan, Albania, a dental community cabinet (Sorriso & Benessere—Ricerca e Clinica SRL, Bari, Italy), the University of Bari Aldo Moro (Italy), and the University of Foggia (Italy).

To perform this study, we recruited 70 pediatric patients from the pediatric and orthodontic private practice clinics. The Institutional Ethics Committee of the Faculty of Technical Medical Sciences of Elbasan “Aleksandër Xhuvani” approved the application to conduct the clinical trial in the faculty (protocol identification: INTL_ALITCOOP/DentPath/2020_SLK). Informed consent was obtained from all subjects' parents or guardians involved in the study. Written informed consent was obtained from the patients' parents or guardians to publish this paper.

The inclusion criteria were as follows:

- Age from 3 to 15 years, with a mean age of 10.4 years, a standard deviation of 2.75, and a median (value that leaves 50% on the left and 50% on the right of a series of data sorted in a nondecreasing way) of 10
- Gender: both male and female
- The absence of situations requiring special diets

The exclusion criteria include all situations that required specific diets, including diabetes and hereditary metabolic diseases such as phenylketonuria, glycogenosis, galactosemia, celiac disease, severe food intolerances, and chronic inflammatory bowel diseases.

During the first phase of the study, we administered a questionnaire to patients that inquired about their eating habits. To obtain more reliable answers, we typically asked for the help of the parents. For the purposes of this research, the software freely available online, following registration at the website http://www.osservatorio.granapadano.it (OGP), was used (i) to evaluate if the patients' food intake was appropriate and (ii) to provide them personalized nutritional advice, highlighting any nutritional deficiencies or excesses caused by their ordinary diet [19].

The first phase, that of the interview, allowed an active healthcare professional- (dentist) patient relationship, strengthened by the emotional elements linked to nutrition (nourishing means, first of all, “taking care” of someone), and the subsequent phase of delivery of the press of the nutritional advice involved communicating things never said before, and do this in a way where the patient feels empathy from the healthcare professional, facilitated by the “counseling” approach. Through this, thanks to the healthcare professional, the patient becomes the protagonist of the change process, acquiring awareness of their own eating habits and lifestyle. This was a useful and powerful tool of improving compliance and achieving expected results more easily.

For the calculation of nutritional needs, the software refers to age groups expressed as follows:

- From 3 to 6 years, male and female
- From 7 to 10 years, male and female
- From 11 to 15 years, male and female
- From 11 to 15 years, female

Data collection was conducted with the help of an electronic questionnaire to evaluate the frequency of weekly or, alternatively, monthly intake of the most common foods of the interviewee's diet, whose nutrient content was “weighed.”

The data collected by the questionnaire were as follows:

- Essential personal data for the inclusion of the subject within a specific cluster
- The weight and height for calculating the body mass index (BMI)
- The abdominal circumference (the weight, height, and abdominal circumference data can be reported by the subject or detected by the healthcare professional, and the software automatically checks “therefore if the healthcare professional decides to detect the data, the corresponding item must be ticked”)
- Lifestyle data (hours spent close to television and/or at personal computer/notebook for free time, smoking habits, physical activity, etc.)

Before moving on to the analysis of individual foods, we asked for the weekly frequency of intake of certain food families (side dishes, fruits, dairy products, meats, and salami). The collected data were compared with those collected during the administration of the detailed questionnaire, in order to reduce the risk of underestimating/overestimating the consumption of relevant foods.

During the detailed interview, a number was inserted for each food that responds to weekly or monthly assumptions; if the food in question was not taken, the corresponding space was left blank. The single intake refers to the quantity of food, expressed in grams, which represents the standard portion for age and sex, which was automatically extrapolated from the dedicated software. When the questionnaire reports more than one food in the same row (for example, beans, lentils, fava beans, chickpeas, and boiled/canned cooked soybeans), it was necessary to sum the intake of the individual foods and report their total value in the box with the relative frequency.

Once the data were acquired through the questionnaire, it was possible to proceed to the oral cavity specialist pathological anamnesis. We asked patients if they suffered from particular mucous diseases, almost always not detectable at the time of the visit, as in the case of vesicular-bullous lesions or aphthous lesions. We investigated the onset of any burning or painful symptoms associated with the ingestion of particular foods and the possible remission of the symptoms when avoiding the intake.

The third step consisted of the objective examination of the patients' oral cavity to assess the presence of caries, earlier restorative treatments, endodontic therapies, dental erosions, missing teeth, enamel hypoplasia, and calcifications, as well the gingival status and the presence of plaque/calculus.

Therefore, the information available for each patient included the following:

- The nutritional status
- The nutritional deficiencies or excesses
- The clinical history
- The current presence of caries, already restored teeth, enamel hypoplasia, enamel erosion, gingivitis, and periodontal disease
- Information reported on the possible appearance of aphthous lesions

We collected the data acquired through the OGP software, the anamnesis, and the physical examination, and, in particular, for each of the 70 patients, we assessed the presence or absence of the following:

- Excesses of proteins, carbohydrates, lipids, saturated fats, and cholesterol
- Deficiencies of carbohydrates, lipids, vitamin C, vitamin A, vitamin D, calcium, iron, and omega 3
- The presence of four types of lesions: caries, gingivitis, enamel hypoplasia, and aphthosis

The above elements were the variables available to us to conduct a study on the association between nutritional alterations and oral lesions.

The statistical analysis between the variables was performed with the chi-square test (*χ*^2^) for the evaluation of the significance of the association or independence. The degree of association between the nominal variables was verified with Cramér's V index (*C*).

## 3. Results

The results showed the following deficiencies in the diet (expressed as percentages): calcium (67%), vitamin D (66%), iron (56%), vitamin A (30%), carbohydrates (23%), omega 3 (20%), lipids (17%), and vitamin C (16%). The excessive amounts of nutrients introduced with diet were cholesterol (64%), saturated fats (63%), proteins (58.56%), lipids (44%), and carbohydrates (20%) (Tables 1(a)–1(c)).

As reported in Table 2, it shows the average ages, and most of the alterations (9 out of 13) were present at a slightly lower average age. The excesses of carbohydrates and saturated fats and the deficiencies of carbohydrates, lipids, vitamin C, vitamin D, calcium, iron, and omega 3 were present in subjects with a lower average age compared with those who were not characterized by such alterations. Only the excesses of proteins, lipids, and cholesterol and deficiency of vitamin A were found in patients with a higher average age compared to patients who did not have these alterations.

From this, we consider that the diet tended to improve with advancing growth. Clearly, we are talking about children who, having an age greater than 3 years, begin to select foods of their preference, avoiding quality (Table 3).

Regarding the sample's oral lesions, the following were found:

- 50% had caries
- 34% had periodontal disease, represented by mild or moderate gingivitis
- 33% had hypoplasia of the enamel
- 20% reported the more or less frequent onset of canker sores

The rejection region for the *χ*^2^ test of independence (Table 4), between the explanatory variable (presence or absence of nutritional alteration) and dependent variable (presence or absence of a lesion), showed dependence between the following:

- Caries and excess lipids and carbohydrates
- Periodontal disease and excess saturated fats and carbohydrates
- Periodontal disease and deficient vitamin C and iron
- Enamel hypoplasia and deficient vitamin D
- Aphthae and deficient vitamin A

Through Cramér's V index, these associations were classified based on their degree of significance (Table 5).

1. Vitamin A deficiency and canker sores (*C* = 0.37)
2. Excess carbohydrates and caries (*C* = 0.35)
3. Vitamin C deficiency and periodontal disease (*C* = 0.34)
4. Iron deficiency and periodontal disease (*C* = 0.28)
5. Excess lipids and caries (*C* = 0.258)
6. Excess of saturated fat and periodontal disease (*C* = 0.254)
7. Carbohydrate deficiency and periodontal disease (*C* = 0.251)
8. Vitamin D deficiency and enamel hypoplasia (*C* = 0.24)

## 4. Discussion

The results obtained in this study regarding the association between nutritional alterations and oral manifestations have found confirmation in various studies [19–21]. Several studies have shown the existence of an association between nutritional alterations and tooth decay: studies that have evaluated the distribution of the body mass index (BMI) and the D(3+4)MFT index in a sample of children and have compared the different regression models by analyzing the association between these two indices [22–24]. Chen et al. in a cross-sectional study stated that an excess of fats does not predispose to caries [25], data confirmed by subsequent studies [26–28].

Bowen, on the other hand, reported how the significant presence of fats in the diet influenced cariogenicity, as fats would increase the clearance of sugars in the oral environment. It would also be conceivable that many fatty acids would exhibit a powerful antibacterial effect [24]; also, changes in the lipid levels and fatty acid composition could, therefore, be associated with caries development [29–32]. Earlier studies confirmed the association between caries and a sugar diet in accordance with the results of our study [33–36]. Gerdin et al. in a study aimed at evaluating the association between dental caries, body mass index, and socioeconomic status in Sweden concluded that the prevalence of being overweight and having caries was (weakly) associated in Swedish children [33].

Murty et al. reported in their study that saliva played a very important role in the prevention and development of tooth decay in enamel [35]. The study involved a comparison between two samples, one with caries-resistant subjects and the other with caries-sensitive subjects, showing that lipid concentration in the parotid saliva was higher in subjects with caries susceptibility [35].

Special cases are represented by the baby bottle syndrome: in the context of pathologies affecting deciduous teeth, we refer to the baby bottle tooth decay syndrome, which derives from incorrect use of the bottle [37–39]. For instance, letting the child fall asleep with the bottle by sucking milk or other sweet substances, or leaving the child for hours with a pacifier soaked in cariogenic substances (i.e., honey or sugar), is strongly discouraged as it often causes the formation of caries so extensive as to reduce the tooth to a small dark stump with consequent, painful abscesses, leading in this way to extracting the decayed dental elements. Statistically, children under 3 years of age are the most affected (in particular the incisor area) [40–43].

For the association between canker sores and vitamin A deficiency, the control function of vitamin A in the keratinization, maturation, and hydration of mucous membranes and skin was implicated [44–47]. Various studies confirm the association between aphthosis and nutritional deficiencies; however, they principally concerned deficiencies of iron, folate, and vitamins of the B group [48–51].

In the literature, we did not find clinical studies in which the association between the onset of aphthosis and the low vitamin A diet was highlighted [52–55]. In our findings, this association was established. Only Scully and Boyle describe, in 1992, in a review, the role of vitamin A in the prevention of potentially malignant lesions by indicating the protective effects but do not describe their pathogenic mechanisms [56].

Associations between vitamin C and D deficit and periodontal disease are described in the literature [56–59]. In our study, only four patients demonstrated a deficit of vitamin C (contained deficit), but all had a sustained degree of gingivitis [60, 61]. These were patients who had undergone more than one oral hygiene session due to the increased susceptibility to plaque buildup and inflammatory responses of the gums that led to edema and bleeding on probing more easily [62–64].

Regarding the association between a high-fat diet and periodontal disease, we can reiterate that, as adipose tissue is a source of inflammatory cytokines, an increase in body fat increases the risk of an increased inflammatory response in periodontitis [65, 66]. Several studies have shown the association between obesity and periodontitis [67–69].

Obesity is characterized by abnormal or excessive deposition of fat in the adipose tissue. The consequences go far beyond negative metabolic effects on health, causing an increase in oxidative stress, which leads not only to endothelial dysfunction but also to negative effects in relation to periodontitis, due to the increase in the inflammatory cytokines that are produced [70]. Thus, obesity appears to participate in the multifactorial phenomenon of the causality of periodontitis through an increase in the production of reactive oxygen species [70, 71].

Another association that emerged from our study was between a low-iron diet and periodontal disease. Enhos et al. led a study aimed at assessing the periodontal health status in patients with iron deficiency anemia, through the detection of ferritin levels in the crevicular fluid before and after periodontal therapy and concluded that iron deficiency was not a factor of direct risk for periodontal disease. There are, however, many other lesions associated with iron deficiency, such as atrophy of the lingual papillae, atrophic glossitis, angular cheilitis, and hyposalivation [72].

Regarding the association we found between periodontal disease and the low introduction of carbohydrates, the literature does not provide us with much data. Merchant et al.'s study showed an inverse correlation between the intake of whole grains and periodontitis [73]. Finally, regarding the last association between vitamin D deficiency and enamel hypoplasia, we can confirm this strong association widely described in the literature [74]. Vitamin D is strongly implicated as it is a direct protagonist of the deposition of calcium and phosphorus in the bones and teeth [75–78].

Throughout physical/oral examinations and the study of each patient's medical records, we collected data regarding the presence of oral lesions.

Statistical analysis confirmed many associations already reported in literature so far, introducing new original data. In this study, being a preliminary study, the results can be used as a baseline data for future studies with similar study design. For the issues related to “excess carbohydrates and caries,” “vitamin C deficiency and periodontal disease,” and “vitamin D deficiency and enamel hypoplasia,” the associations already established were confirmed [79–82]. Therefore, in these three specific investigations, our findings have the role only to strengthen and confirm current concepts. In contrast, the issues related to “vitamin A deficiency and canker sores,” “iron deficiency and periodontal disease,” and “carbohydrate deficiency and periodontal disease,” more attractive results are introduced. In particular, association between “vitamin A deficiency and canker sores” not only confirmed but also showed a higher statistical significance. This could be due to the role that vitamin A plays in epithelialisation and keratinisation.

For the last association related to iron-periodontopathic deficiency, with regard to points “excess lipids and caries” and “excess of saturated fats and presence of periodontal disease,” the relationships are according to literature reports [83–85]. However, studies of longer duration with cross-over study design and wider sample size would have been more authenticating as it eliminates the bias of viable host.

## 5. Conclusions

The most relevant data emerging from the current research was the presence of an association between vitamin A deficiency and canker sores (*C* = 0.37) that has been not often described in scientific literature. The obtained results lead us to conclude that a correct and balanced diet, without excess and without nutritional deficiencies, contributes to the well-being of overall human body, including oral cavity, laying the fundamentals for proper tooth structure, maintaining the tropism of the mucosal membranes.

In addition, conclusions on an association of nutrient deficiency with periodontal disease cannot be assessed if the results are adjusted for comorbidity, such as smoking, diabetes mellitus, and other systemic diseases.

To produce conclusive evidence on the subject of this study, longitudinal cohort studies and follow-up randomized controlled trials are needed.

## Figures and Tables

**Table:** (a) Population and distribution of subjects (total = 70), with an excess of each of the variables considered

Excess	*n*	%
Protein	41	58.6
Lipids	31	44.3
Carbohydrates	14	20.0
Saturated fats	44	62.9
Cholesterol	45	64.3

**Table:** (b) Population and distribution of subjects (total = 70), with a deficiency of each of the variables considered

Deficiency	*n*	%
Carbohydrates	16	22.9
Lipids	12	17.1
Vitamin C	4	5.7
Vitamin A	21	30.0
Vitamin D	46	65.7
Calcium	47	67.1
Iron	39	55.7
Omega 3	14	20.1

**Table:** (c) Population and distribution of subjects (total = 70), with a deficiency of each of the variables considered

Oral lesions	*n*	%
Caries	35	50.0
Gingivitis	24	34.2
Hypoplasia	23	32.9
Aphthae	14	20.0

**Table:** (a) Ratio of the number of individuals with the presence (P) or absence (A) of excesses for each nutritional variable into the sample size (70 patients). Upper (U.L.) and lower (L.L.) limits can differ, within the sample population

Excess	A (*n*)	P (*n*)	U.L.	L.L.
Protein	0.41	0.59	0.70	0.47
Lipids	0.56	0.44	0.56	0.33
Carbohydrates	0.80	0.20	0.29	0.11
Saturated fats	0.71	0.63	0.74	0.52
Cholesterol	0.36	0.64	0.76	0.53

**Table:** (b) Ratio of the number of individuals with the presence (P) or absence (A) of deficiencies for each nutritional variable into the sample size (70 patients). Upper (U.L.) and lower (L.L.) limits can differ, within the sample population

Deficiency	A (*n*)	P (*n*)	U.L.	L.L.
Carbohydrates	0.77	0.23	0.33	0.13
Lipids	0.83	0.17	0.26	0.08
Vitamin C	0.94	0.06	0.14	0.02
Vitamin A	0.70	0.30	0.41	0.19
Vitamin D	0.34	0.66	0.77	0.55
Calcium	0.33	0.67	0.78	0.56
Iron	0.44	0.56	0.67	0.44
Omega 3	0.80	0.20	0.29	0.11

**Table:** (c) Ratio of the number of individuals with the presence (P) or absence (A) of specific oral lesions to the sample size (70 patients). Upper (U.L.) and lower (L.L.) limits can differ, within the sample population

Oral lesions	A (*n*)	P (*n*)	U.L.	L.L.
Caries	0.50	0.50	0.62	0.38
Gingivitis	0.66	0.34	0.45	0.23
Hypoplasia	0.67	0.33	0.44	0.22
Aphthae	0.80	0.20	0.29	0.11

**Table 3:** Patients' mean age in the presence (P) or absence (A) of each nutritional alterations and oral lesions. The table also highlights whether the alteration characterizes a trail of greater or lower age (*P* major/minor).

	A	P	*P* major/minor
Excess			
Protein	9.93	10.12	+0.19
Lipids	9.31	10.97	+1.66
Carbohydrates	10.09	9.86	-0.23
Saturated fats	10.08	10.02	-0.05
Cholesterol	9.92	10.11	+0.19
Deficiency			
Carbohydrates	10.09	9.88	-0.22
Lipids	10.26	9.00	-1.26
Vitamin C	10.18	7.75	-2.43
Vitamin A	9.98	10.19	+0.21
Vitamin D	10.42	9.85	-0.57
Calcium	10.48	9.83	-0.65
Iron	10.10	10.00	-0.10
Omega 3	10.57	7.93	-2.64
Oral lesions			
Caries	10.20	9.89	-0.31
Gingivitis	9.98	10.17	+0.19
Hypoplasia	10.11	9.91	-0.19
Aphthae	10.05	10.00	-0.05

**(a):** 

Excess	Protein	Lipids	Carbohydrates	Saturated fats	Cholesterol
Caries	0.0589	4.6898^∗^	8.9286^∗^	0.2448	0.5600
Gingivitis	0.2323	0.0355	0.2536	4.5335^∗^	0.0507
Hypoplasia	3.3226	0.3690	1.0361	0.0580	0.0130
Aphthae	0.5299	1.7514	1.8080	0.0153	0.0000

**(b):** 

Deficiency	Carbohydrates	Lipids	Vit. C	Vit. A	Vit. D	Calcium	Iron	Omega 3
Caries	0.0000	3.6207	1.0606	0.0680	2.2826	1.6189	2.8371	3.2143
Gingivitis	4.4410^∗^	0.3502	8.1313^∗^	0.4348	1.3977	0.0038	5.5054^∗^	0.5707
Hypoplasia	0.5804	0.0015	0.5651	0.2498	4.3395^∗^	1.9193	0.1740	0.0648
Aphthae	1.6406	1.6092	0.0663	9.7959^∗^	0.0159	1.0361	3.7055	2.7009

**(a):** 

Excess	Protein	Lipids	Carbohydrates	Saturated fats	Cholesterol
Caries	0.0290	0.2588^∗^	0.3571^∗^	0.0591	0.0894
Gingivitis	0.0576	0.0225	0.0602	0.2545^∗^	0.0269
Hypoplasia	0.2179	0.0726	0.1217	0.0288	0.0136
Aphthae	0.0870	0.1582	0.1607	0.0148	0.0000

**(b):** 

Deficiency	Carbohydrates	Lipids	Vit. C	Vit. A	Vit. D	Calcium	Iron	Omega 3
Caries	0.0000	0.2274	0.1231	0.0312	0.1806	0.1521	0.2013	0.2143
Gingivitis	0.2519^∗^	0.0707	0.3408^∗^	0.0788	0.1413	0.0073	0.2804^∗^	0.0903
Hypoplasia	0.0911	0.0046	0.0899	0.0597	0.2490^∗^	0.1656	0.0499	0.0304
Aphthae	0.1531	0.1516	0.0308	0.3741^∗^	0.0150	0.1217	0.2301	0.1964

## Data Availability

The data used to support the findings of this study are included within the article.
